# A Fault Diagnosis Methodology for Gear Pump Based on EEMD and Bayesian Network

**DOI:** 10.1371/journal.pone.0125703

**Published:** 2015-05-04

**Authors:** Zengkai Liu, Yonghong Liu, Hongkai Shan, Baoping Cai, Qing Huang

**Affiliations:** 1 College of Mechanical and Electrical Engineering, China University of Petroleum, Qingdao, 266580, China; 2 Department of Mechanical Engineering, Xi’an Jiaotong University, Xi’an, 710049, China; 3 China Beijing Petrochemical Engineering Co., Ltd, Beijing, 100107, China; Southwest University, CHINA

## Abstract

This paper proposes a fault diagnosis methodology for a gear pump based on the ensemble empirical mode decomposition (EEMD) method and the Bayesian network. Essentially, the presented scheme is a multi-source information fusion based methodology. Compared with the conventional fault diagnosis with only EEMD, the proposed method is able to take advantage of all useful information besides sensor signals. The presented diagnostic Bayesian network consists of a fault layer, a fault feature layer and a multi-source information layer. Vibration signals from sensor measurement are decomposed by the EEMD method and the energy of intrinsic mode functions (IMFs) are calculated as fault features. These features are added into the fault feature layer in the Bayesian network. The other sources of useful information are added to the information layer. The generalized three-layer Bayesian network can be developed by fully incorporating faults and fault symptoms as well as other useful information such as naked eye inspection and maintenance records. Therefore, diagnostic accuracy and capacity can be improved. The proposed methodology is applied to the fault diagnosis of a gear pump and the structure and parameters of the Bayesian network is established. Compared with artificial neural network and support vector machine classification algorithms, the proposed model has the best diagnostic performance when sensor data is used only. A case study has demonstrated that some information from human observation or system repair records is very helpful to the fault diagnosis. It is effective and efficient in diagnosing faults based on uncertain, incomplete information.

## Introduction

As the key element of hydraulic system, a pump is responsible for the mechanical to hydraulic energy conversion process. Gear pumps are widely applied in modern industry owing to its advantages such as small and compact design, high efficiency and low manufacturing cost. The working status of a gear pump greatly affects the performance of the whole hydraulic system, thus it is necessary to develop its fault diagnosis technique. The condition of mechanical equipment is closely associated with vibration signals, which come about during rotating process for rotating machinery [1]. Therefore, fault diagnosis of gear pumps can be performed with the characteristic information extracted by the signal processing techniques such as short time Fourier transform [2], wavelet transform [3], blind source separation [4], sparse decomposition method [5] and empirical mode decomposition (EMD) [6].

EMD is a time-frequency signal processing technique. Compared with other signal processing methods, EMD is self-adaptive and especially suits for the non-stationary and non-linear signals. For sparse decomposition method, the algorithm is much more demanding and complex compared to EMD [7]. Based on the local characteristic time scales of a signal, the original vibration signal can be decomposed into several intrinsic mode functions (IMFs). Due to the adaptive analysis and high robustness nature, EMD has been widely applied in fault diagnosis of rotating machinery [8, 9]. However, EMD suffers from the mode mixing problem, which means a single IMF either consisting of widely disparate scales, or a signal of a similar scale residing in different IMF components [10]. In order to alleviate the problem of mode mixing in EMD, Wu and Huang [11] propose ensemble empirical mode decomposition (EEMD) method. Essentially, white noise of finite amplitude is added to the original signal during the EEMD decomposition process. The ensemble means of the corresponding IMFs generated from each trial are defined as the true IMFs of the EEMD [12]. Lei et al [13] employ EEMD in diagnosing rub-impact faults in a power generator and a heavy oil catalytic cracking machine set. Compared with EMD method, it is demonstrated that EEMD has superiority in fault diagnosis of rotating machinery. Caesarendra et al. [14] apply EEMD method in two real cases of slow speed slewing bearing with natural bearing fault damage and the results show that EEMD is better than FFT in identifying fault frequencies. Mahgoun et al. [15] present the application of EEMD in purpose to detect localized faults of damage at an early stage.

Although previous research jobs on rotating machinery have produced significant outcomes, only information from sensor measurement is used for fault diagnosis. However, other sources of information besides sensor measurement could be useful for fault diagnosis, which are obviously not fully utilized. For example, the observation information from human or the maintenance record would make the diagnosis results more reliable. Obviously, the more evidences are used, the more accurate the diagnostic results will be. Recently, multi-source information fusion fault diagnosis systems based on Bayesian networks have been developed in some fields. To take advantage of all useful information, a three-layered Bayesian network has been presented for fault diagnosis of a chiller [16] and variable air volume terminals [17]. Xu [18] develops an intelligent expert system of rotating flexible rotors based on Bayesian network by fully incorporating human experts’ knowledge, machine faults and faults symptoms as well as machine running conditions. Cai et al. [19] propose a multi-source information fusion based fault diagnosis methodology for ground-source heat pump by making use of sensor information and observation information. Oukhellou et al. [20] present a hybrid diagnosis system based on the combination of local sensor data information and global structural knowledge information for the detection of broken rail.

Bayesian network is an acyclic directed graph consisting of a set of variables with directed edges between the variables. It is a powerful tool for modeling complex problems in probabilistic knowledge representation and reasoning [21]. It has been widely used for fault diagnosis in a variety of fields. Sun et al. [22] develop a Mild Congnitive Impairment (MCI) expert system based on Bayesian network to address MCI’s prediction and inference question and the experimental results indicate that the proposed model achieved better results than some existing methods in most instances. Barco et al. [23] propose a discrete Bayesian network for diagnosis of radio access networks of cellular systems and the research shows that the developed model outperform traditional Bayesian network when there is inaccuracy in the model parameters. Sahin et al. [24] develop a Bayesian network for fault diagnosis of airplane engines and the results show that the proposed model can detect the anomalies or faults in the sensor readings. Kariv et al. [25] develop a computerized decision support system for the diagnosis of infections among solid organ transplant recipients based on Bayesian network.

In this paper, a fault diagnosis methodology for gear pumps based on EEMD method and Bayesian network is proposed to make full use of multi-source information. One of the weak points of Bayesian network is that there is no specific semantic to guide the model development [26].This paper presents a three-layered diagnostic Bayesian network for model development, which is composed of fault layer, fault feature layer and multi-source information layer. With the proposed framework, Bayesian network for fault diagnosis can be easily developed. Vibration signals from sensor measurement are decomposed by the EEMD method and the features of IMFs are extracted. The obtained fault features and other multi-sources information are entered into fault feature layer and multi-source information layer, respectively. The reminder of this paper is organized as follows. In Section 2, EEMD method and Bayesian network are introduced. Section 3 proposes the fault diagnosis methodology and applies it to a gear pump. In Section 4, fault diagnosis based on the developed model is performed. Section 5 summarizes the paper.

## EEMD and Bayesian Network

### EEMD algorithm and feature extraction method

EEMD is proposed to overcome the mode mixing problem, which is defined as a single IMF including oscillations of dramatically disparate scales or a component of a similar scale residing in different IMFs. Essentially, white noise of finite amplitude is added to the original signal during the EEMD decomposition process. In fact, to make the EEMD effective, the amplitude of the added noise should not be too small. In most cases, white noise of an amplitude that is about 0.2 standard deviation of that of the data is suggested. However, when the data is dominated by high-frequency signals, the noise amplitude may be smaller, and when the data is dominated by low-frequency signals, the noise amplitude may be increased. Generally speaking, the range of standard deviation is 0.1–0.4 [11]. The ensemble means of the corresponding IMFs generated from each trial are defined as the true IMFs of the EEMD. The EEMD performance of overcoming the mode mixing problem has been demonstrated [13, 27].

The EEMD algorithm can be given as follows [15].

1. Determine the number of ensemble M and initialize the amplitude of the added white noise, and m = 1.
2. Perform the *m*th trial on the investigated signal added white noise. Add a white noise series with the given amplitude to the original signal.
  $$x_{m}(t)=x(t)+n_{m}(t)$$ (1)
  where *n*_*m*_(*t*) represents the *m*th added white noise series and *x*_*m*_(*t*) denotes the noise-added signal of the *m*th trial.
3. With the EMD method [28], the noise-added signal *x*_*m*_(*t*) is decomposed into N IMFs *c*_*n*,*m*_(*t*)(*i*=1,2,…,I), where *c*_*n*,*m*_(*t*) represents the *n*th IMF of the *m*th trial, and N is the number of IMFs.
4. If *n* < *M* then let m = m + 1. Repeat steps (2) and (3) again and again with different white noise series each time until *n* = *M*.
5. Calculate the ensemble mean *c*_*n*_(*t*) of the M trials for each IMF
  $$a_{i}(t)=\frac{1}{M}\sum_{m=1}^{M}c_{n,m},n=1,2,\ldots ,N$$ (2)
6. Report the mean *a*_*i*_(*t*) (i = 1, 2,…, N) of each of the *N* IMFs as the final IMFs.

**Fig 1:**
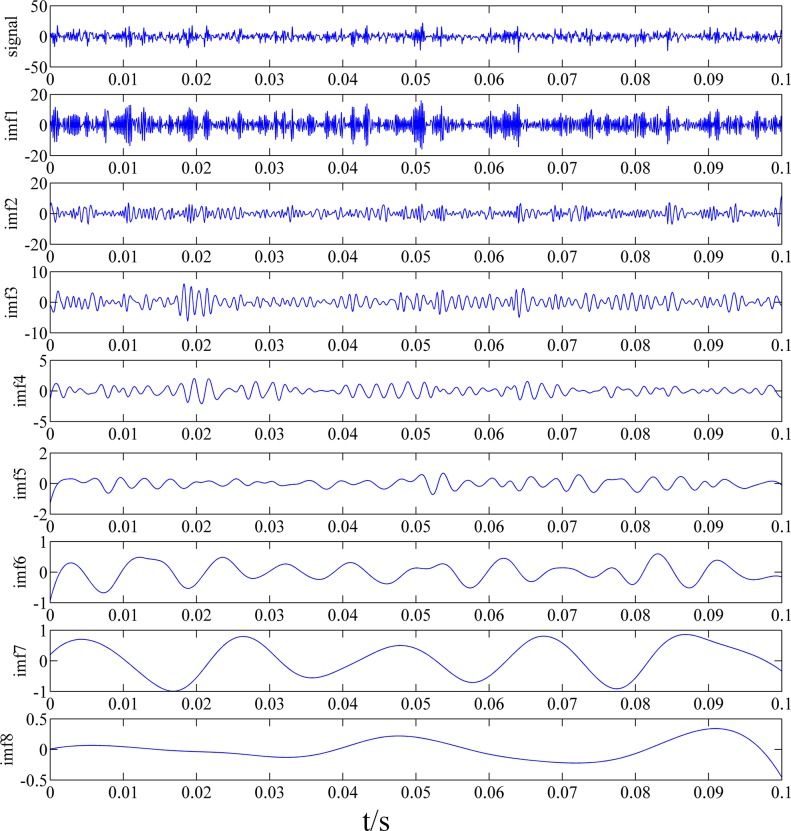
The decomposed components with EEMD and original signal.

According to the above steps, a vibration signal measured from a gear pump is decomposed and the decomposition result is given in Fig 1. It shows 8 IMFs in different frequency bands decomposed by EEMD algorithm. It can be seen from the figure that the original signal is very complicated and the decomposed IMFs are hard to use for fault diagnosis. Hence, features of the signals need to extract.

Feature extraction is an important step for fault diagnosis. IMFs decomposed by EEMD contain valid information for fault diagnosis. The analysis results from EEMD energy of different vibration signals indicate that the energy of a vibration signal will change in different frequency bands when a fault occurs. It means that for the same faults, the decomposed IMFs are similar in the corresponding frequency band. Therefore, the energy of the decomposed IMFs could be used as features for fault diagnosis. *E*_*i*_ is the energy of the *i*th IMF.

$$E_{i}=\int_{-\infty }^{+\infty }|a_{i}(t){|}^{2}dt,i=1,2,3,\ldots n$$ (3)

Then the feature vector of the investigated signal *T* = [*E*_1_, *E*_2_, *E*_3_, … *E*_*n*_] is obtained.

### Bayesian network

Bayesian network is a directed acyclic graph that is composed of a set of variables {*X*_1_, *X*_2_, …, *X*_*N*_} and a set of directed edges between the variables [29, 30]. A variable has several possible states, such as true and false. Bayesian networks are very successful in probabilistic knowledge representation and reasoning. In Bayesian networks, the joint probability distribution function of all nodes can be calculated by Eq. (4).

$$P(X_{1},X_{2},\ldots ,X_{N})=\prod_{i=1}^{N}P(X_{i}|Pa_{i})$$ (4)

Where *Pa*_*i*_ is the set of random variables whose corresponding nodes are parent nodes of *X*_*i*_.

**Fig 2:**
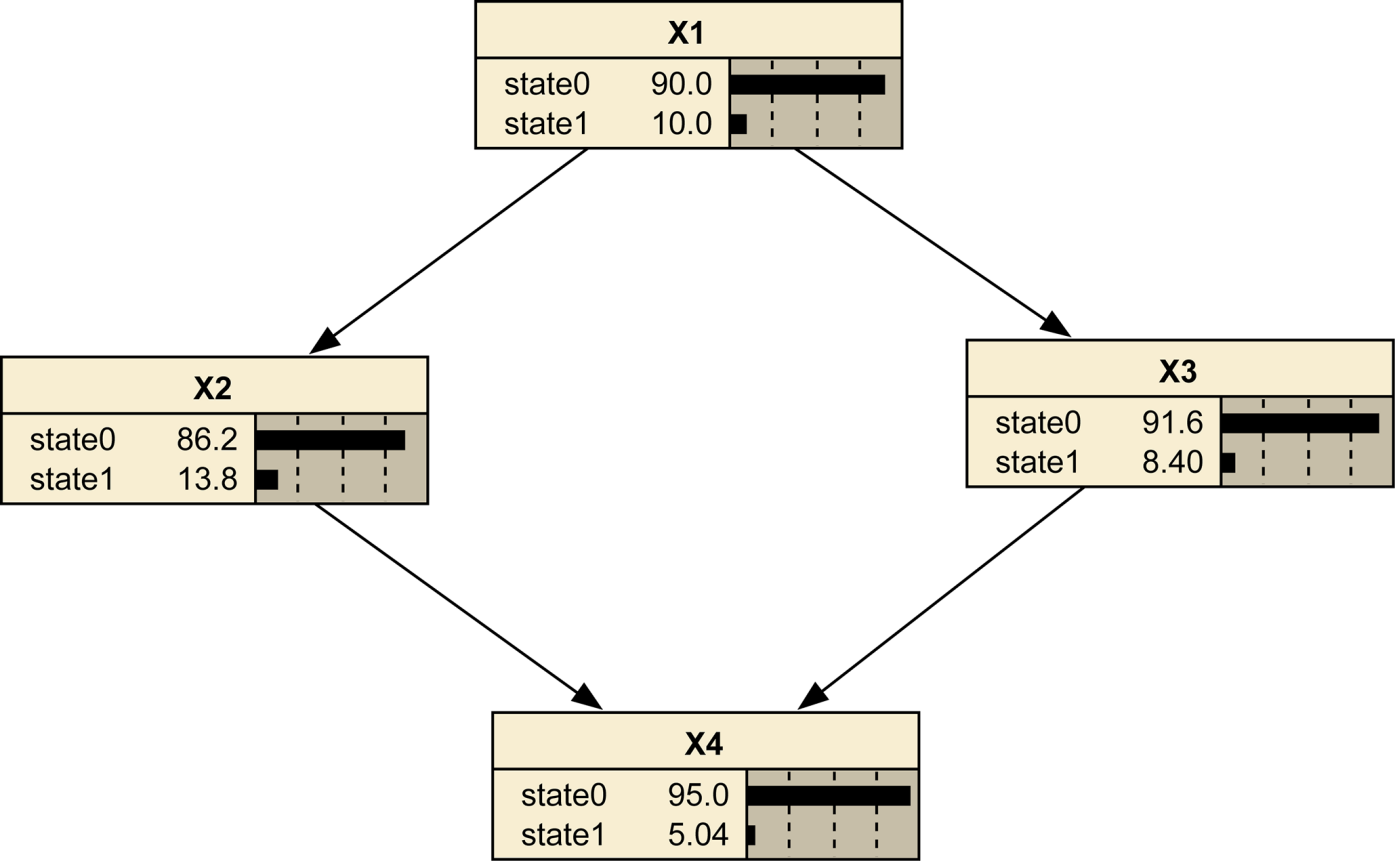
A simple Bayesian network.

A Bayesian network contains two elements, namely structure and parameters. An example shown in Fig 2 is used to illustrate the basic idea of Bayesian networks. In Fig 2, the nodes (*X1*, *X2*, *X3*, *X4*) represent random variables and arcs means dependence relationships among them. Each arc starts from a parent node and ends at a child node. *Pa(X)* represents the parent nodes of node *X*, therefore, *Pa(X2) = {X1}*, *Pa(X3) = {X1}*, *Pa(X4) = {X2*, *X3}*. *X1* is the root node, because it has no input arcs. Each node has two states: state0 and state1. Root nodes have prior probabilities. Each child node has conditional probabilities based on the combination of states of its parent nodes.

### The Proposed Fault Diagnosis Methodology and Its Application

#### The proposed methodology

**Fig 3:**
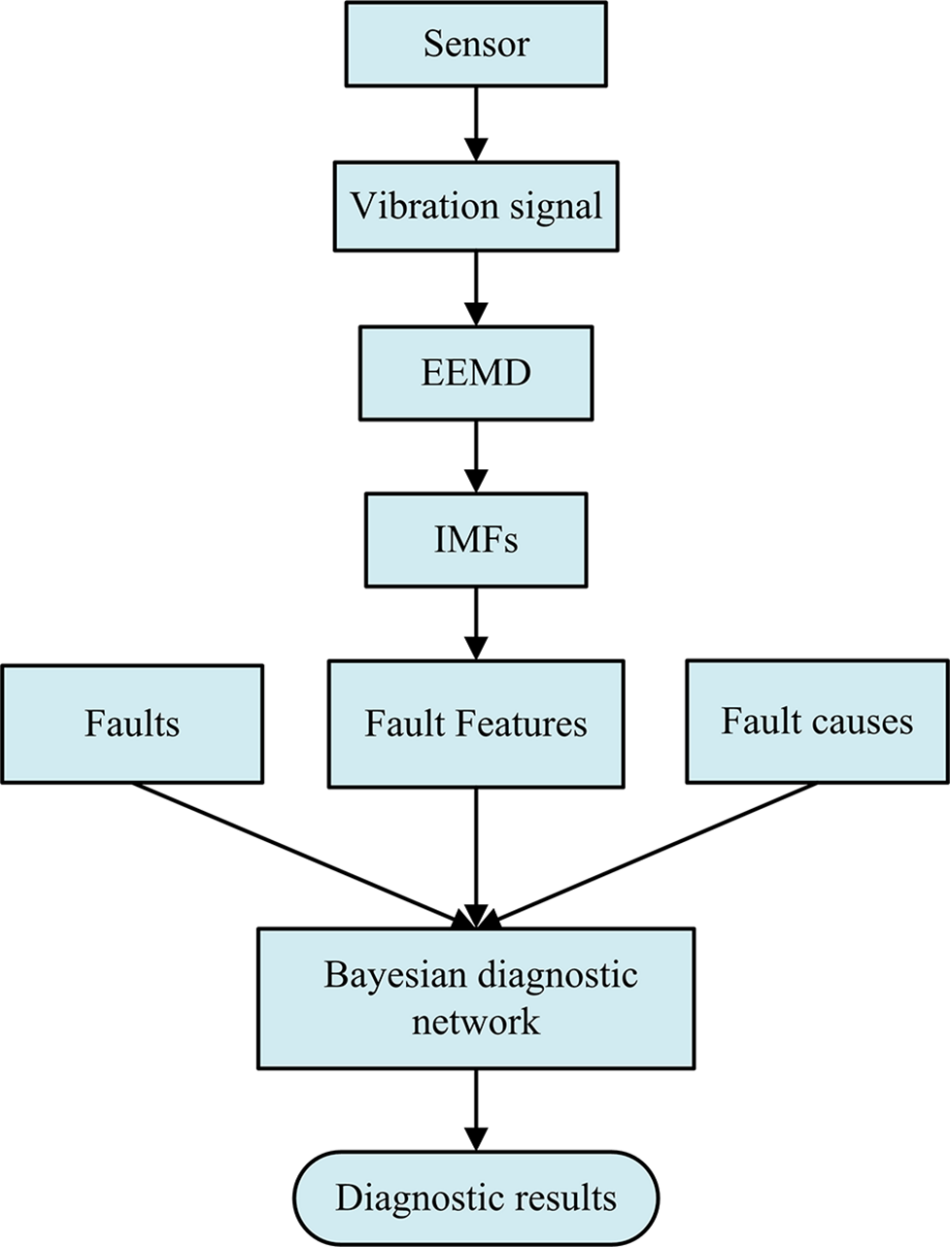
Flow chart of the proposed fault diagnosis methodology.

Fault diagnosis methods in previous research are mainly based on fault features extracted by some signal processing techniques. In this paper a fault diagnosis methodology for gear pump based on EEMD and Bayesian network is proposed. Flow chart of the methodology is shown in Fig 3. To establish the Bayesian network, the investigated faults, fault features from sensor data and multi-source information are integrated into the diagnostic model. According to the evidences obtained from the fault features and multi-source information, a fault of the gear pump could be diagnosed.

The proposed methodology consists of fault detection, signal processing and fault diagnosis. For fault detection, a sensor is responsible for monitoring the vibration signal of the gear pump, and then the data is decomposed by EEMD method to obtain its IMFs. Fault feature extraction is accomplished by calculating the energy of IMFs according to Eq. (3). Actually, the diagnostic model includes three layers: fault layer, fault feature layer and multi-source information layer. Features decomposed by EEMD method will be entered into the fault feature layer. Fault layer includes the common faults to identify. Obviously, the more faults to diagnose, the more complicated the diagnosis system will be. For multi-source information, all the factors such as human observation information, system maintenance information or abnormal operation records are directly related to the probability of occurrence of the faults. For example, tooth face wear in fault layer is less possible to appear if the gear has been replaced by a new one during the recent maintenance.

### Experiment and feature extraction

**Fig 4:**
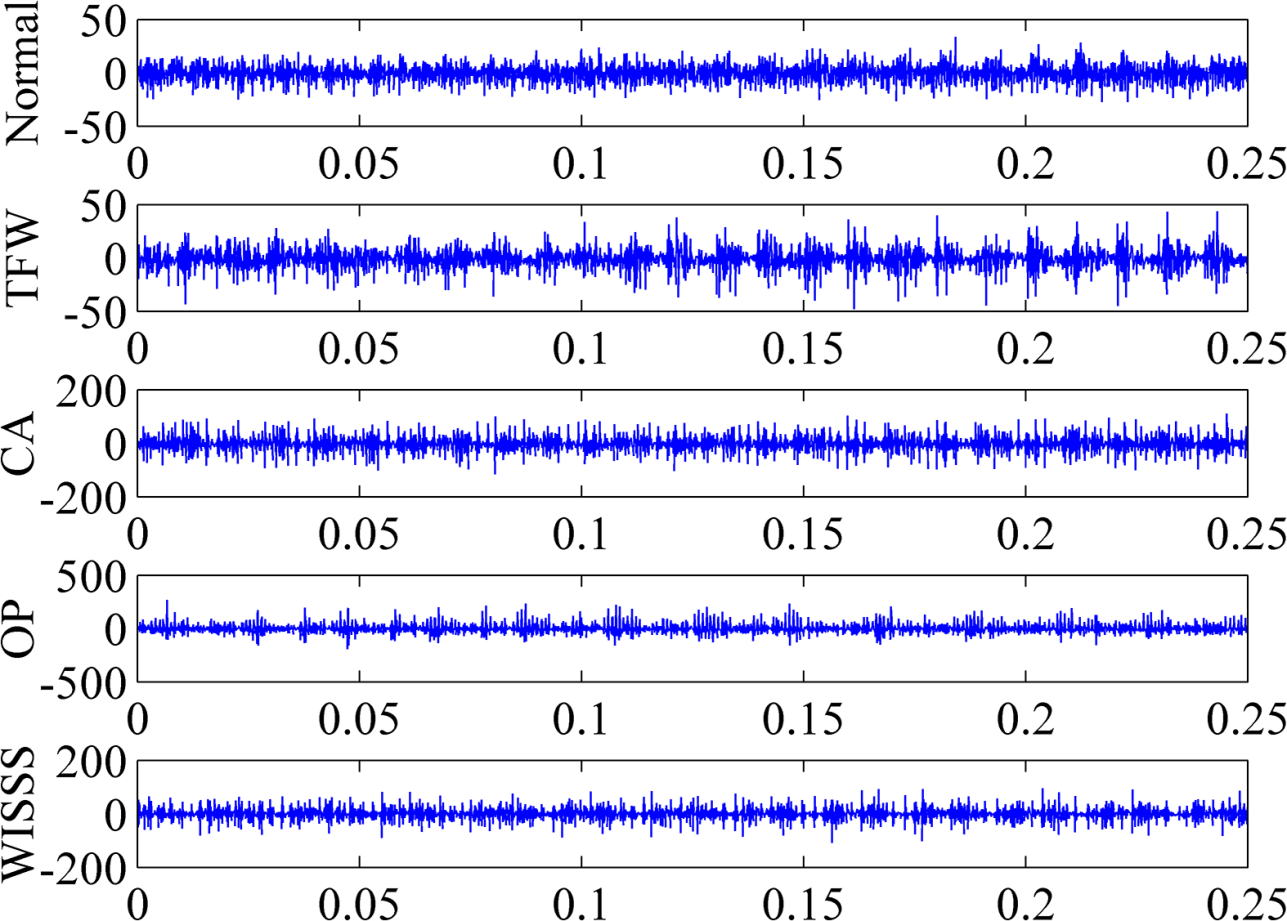
Vibration signals of the gear pump in different conditions.

The proposed methodology is applied to a gear pump with the type of WCB-50. In this paper, four common fault reasons including tooth face wear (TFW), cavitation (CA), oil pollution (OP) and wear of internal surface of shaft sleeve (WISSS) are investigated. To obtain the data sets, these four fault reasons are simulated respectively. TFW is simulated by grinding one of the meshing surfaces of driving gear. CA is simulated by loosening the oil pump inlet. By adding pollutants into the working oil, OP is simulated. WISSS is simulated by grinding the internal surface of shaft sleeve. A piezoelectric acceleration sensor is used for collecting the vibration signal and it is connected to a dynamic test and analysis system with the type of DH5923. The sampling frequency is set as 10 kHz. Each fault has 100 training and 50 testing instances. There are 400 training and 200 testing samples in total. Vibration signals of the gear pump with different faults and in normal condition are plotted in Fig 4.

According to the EEMD algorithm and feature extraction process described in Section 2.1, the vibration signals from different conditions are decomposed. Because the last few IMFs contain very little energy, which are useless for fault diagnosis, only the first 8 IMFs are selected for each signal. Therefore 8 features based on the energy of IMFs are calculated, which are used to identify the faults. Before a feature is entered into the Bayesian network, it is discretized according to the range of values of the data samples. Although increasing the number of intervals can improve the accuracy, it will increase the burden for building the Bayesian network. To balance the accuracy and difficulty of developing the Bayesian network, 6 intervals are determined. After discretization, the extracted feature can be denoted by one of the six numbers (1, 2,…, 6). Table 1 is the training samples of discretized features of four faults. In the table, feature *i* is denoted by *Feai (i = 1*,*2*,*…*,*8)*. The testing samples can be obtained in the same way.

**Table 1:** Training samples of discretized features of four faults.

Samples	Fea1	Fea2	Fea3	Fea4	Fea5	Fea6	Fea7	Fea8	Conditions
1	4	3	5	5	2	2	4	1	TFW
2	4	3	6	6	1	3	5	1	TFW
3	4	3	6	6	2	3	4	2	TFW
⁝	⁝	⁝	⁝	⁝	⁝	⁝	⁝	⁝	⁝
398	6	1	1	2	1	1	2	1	WISSS
399	6	1	2	2	1	1	1	1	WISSS
400	6	1	1	2	1	1	2	1	WISSS

### Bayesian network structure

**Fig 5:**
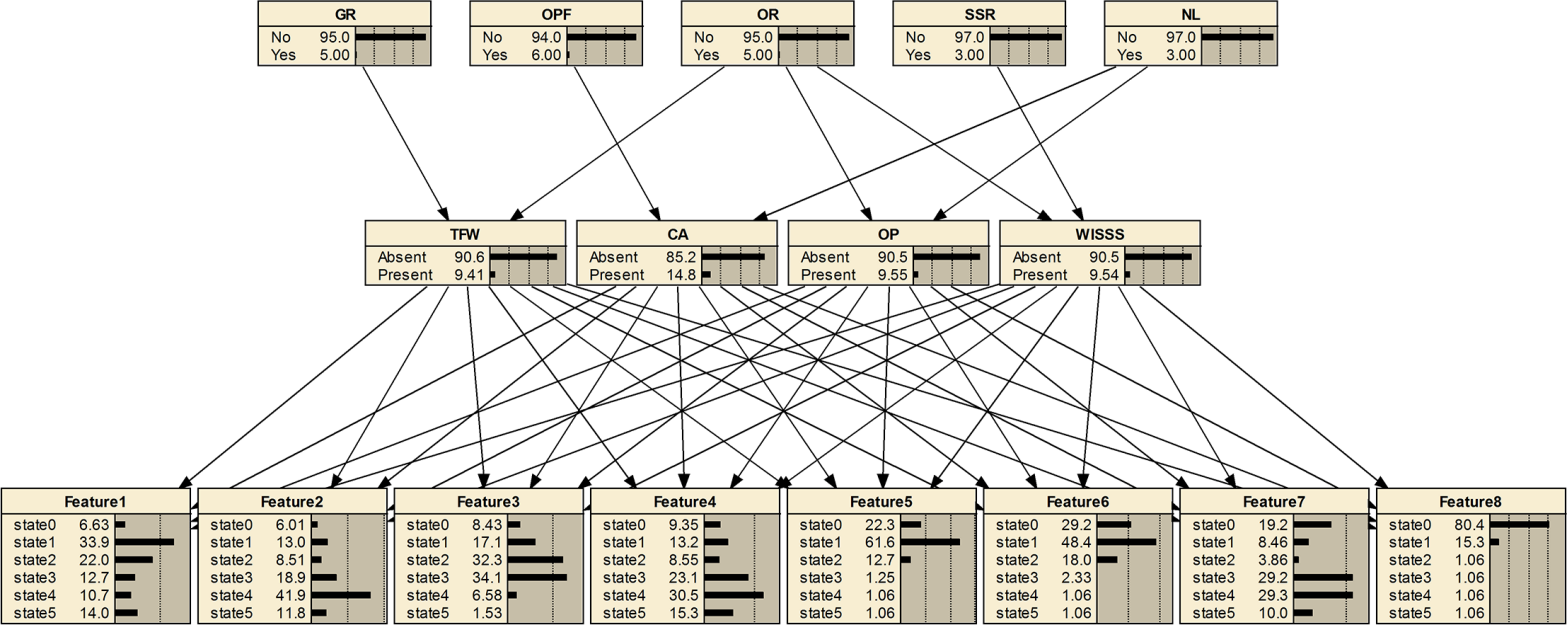
Developed Bayesian network for gear pump.

The structure of the Bayesian network is a graphic illustration about the qualitative relationships nodes in different layers. The Bayesian diagnostic network based on the proposed methodology is shown in Fig 5. The developed Bayesian network has three layers: fault feature layer, fault layer and multi-source information layer. The directed arc denotes that each parent node will cause changes of the child nodes.

The fault layer includes four nodes, indicating the investigated faults. After the nodes are determined, the states of each node should be defined. In the fault layer, each node has two states, namely present and absent, indicating the presence and absence of the fault, respectively. The fault feature layer consists of eight child nodes, indicating eight features extracted from sensor signals using EEMD method. Each feature node has six states (state1-state6), representing its interval that the energy value of IMF belongs to. Multi-source information useful to diagnose the gear pump could be added into the information layer. In this paper, human observation and repair service information of the gear pump are adopted. Five casual factors are selected, namely gear replacement (GR), oil pipe folding (OPF), oil replacement (OR), shaft sleeve replacement (SSR) and noise level (NL).The events that the nodes represent are listed in Table 2. Each node has two states: yes and no.

**Table 2:** Nodes and their states in the multi-source information layer.

Node	Event	State	Prior probability
GR	Is the wear replaced by a new one in the last maintenance?	Yes	5%
		No	95%
OPF	Is the oil pipe of the gear pump folded?	Yes	6%
		No	94%
OR	Is the oil replaced by new one in the last maintenance?	Yes	5%
		No	95%
SSR	Is the shaft sleeve replaced by a new one in the last maintenance?	Yes	3%
		No	97%
NL	Is the noise level high?	Yes	3%
		No	97%

#### Bayesian network parameters

When the structure of Bayesian network is established, the prior probabilities and conditional probabilities are required to specify. A prior probability is the probability that an event occurs without new evidence or information. Usually, prior probabilities are determined by the experts or statistical analysis of historical data. Since historical data is hardly available, prior probabilities are often obtained according to the expert knowledge [16, 17]. It is obvious that the higher the prior probability of an event, the more likely it is to occur. In this paper, prior probabilities of the nodes in the information layer are determined in Table 2.

A conditional probability is the probability that an event occurs for the given new evidence. The conditional probabilities among the nodes in the multi-source information layer and the nodes in the fault layer are set according to the knowledge and experience of authors. They are shown in Tables 3–6.

**Table 3:** Conditional probability table of node TFW.

Fault	GR	YES		NO	
	OR	YES	NO	YES	NO
TFW	Present	0.01	0.03	0.05	0.1
	Absent	0.99	0.97	0.95	0.9

**Table 4:** Conditional probability table of node CA.

Fault	OPF	YES		NO	
	NL	YES	NO	YES	NO
CA	Present	0.95	0.9	0.1	0.08
	Absent	0.05	0.1	0.9	0.92

**Table 5:** Conditional probability table of node OP.

Fault	OR	YES		NO	
	NL	YES	NO	YES	NO
OP	Present	0.07	0.05	0.12	0.1
	Absent	0.93	0.95	0.88	0.9

**Table 6:** Conditional probability table of node WISSS.

Fault	SSR	YES		NO	
	OR	YES	NO	YES	NO
WISSS	Present	0.01	0.03	0.05	0.1
	Absent	0.99	0.97	0.95	0.9

The conditional probabilities of a child node depend on all the possible combination of states of its parents. For instance, Feature 1 in the fault feature layer has four parent nodes. Therefore, it has 96 (6*2^4^) conditional probabilities and the 8 feature nodes need 768 parameters in total. To reduce the number of parameters need to specify conditional probabilities, Noisy-MAX node is applied [31]. The nodes in the fault feature layer are set as Noisy-MAX nodes. Hence, conditional probabilities calculated statistically using the training samples are used as parameters for those Noisy-MAX nodes. They are listed in Table 7.

**Table 7:** The conditional probability among nodes in the fault layer and feature layer.

Fault	State	Fea1	Fea2	Fea3	Fea4	Fea5	Fea6	Fea7	Fea8
TFW	1	0.01	0.01	0.01	0.01	0.17	0.3	0.01	0.76
	2	0.01	0.01	0.01	0.01	0.73	0.61	0.01	0.2
	3	0.13	0.61	0.08	0.07	0.07	0.06	0.11	0.01
	4	0.75	0.35	0.51	0.61	0.01	0.01	0.59	0.01
	5	0.09	0.01	0.33	0.25	0.01	0.01	0.26	0.01
	6	0.01	0.01	0.06	0.05	0.01	0.01	0.02	0.01
CA	1	0.01	0.05	0.26	0.76	0.76	0.95	0.95	0.95
	2	0.01	0.86	0.7	0.2	0.2	0.01	0.01	0.01
	3	0.01	0.06	0.01	0.01	0.01	0.01	0.01	0.01
	4	0.01	0.01	0.01	0.01	0.01	0.01	0.01	0.01
	5	0.65	0.01	0.01	0.01	0.01	0.01	0.01	0.01
	6	0.31	0.01	0.01	0.01	0.01	0.01	0.01	0.01
OP	1	0.01	0.01	0.02	0.03	0.04	0.89	0.95	0.81
	2	0.03	0.01	0.69	0.68	0.59	0.07	0.01	0.15
	3	0.3	0.21	0.26	0.26	0.32	0.01	0.01	0.01
	4	0.57	0.58	0.01	0.01	0.03	0.01	0.01	0.01
	5	0.08	0.18	0.01	0.01	0.01	0.01	0.01	0.01
	6	0.01	0.01	0.01	0.01	0.01	0.01	0.01	0.01
WISSS	1	0.01	0.7	0.68	0.01	0.87	0.93	0.08	0.76
	2	0.01	0.26	0.28	0.6	0.09	0.03	0.88	0.2
	3	0.01	0.01	0.01	0.36	0.01	0.01	0.01	0.01
	4	0.01	0.01	0.01	0.01	0.01	0.01	0.01	0.01
	5	0.01	0.01	0.01	0.01	0.01	0.01	0.01	0.01
	6	0.95	0.01	0.01	0.01	0.01	0.01	0.01	0.01

### Fault Diagnosis and Discussion

#### Fault diagnosis only using fault features

**Fig 6:**
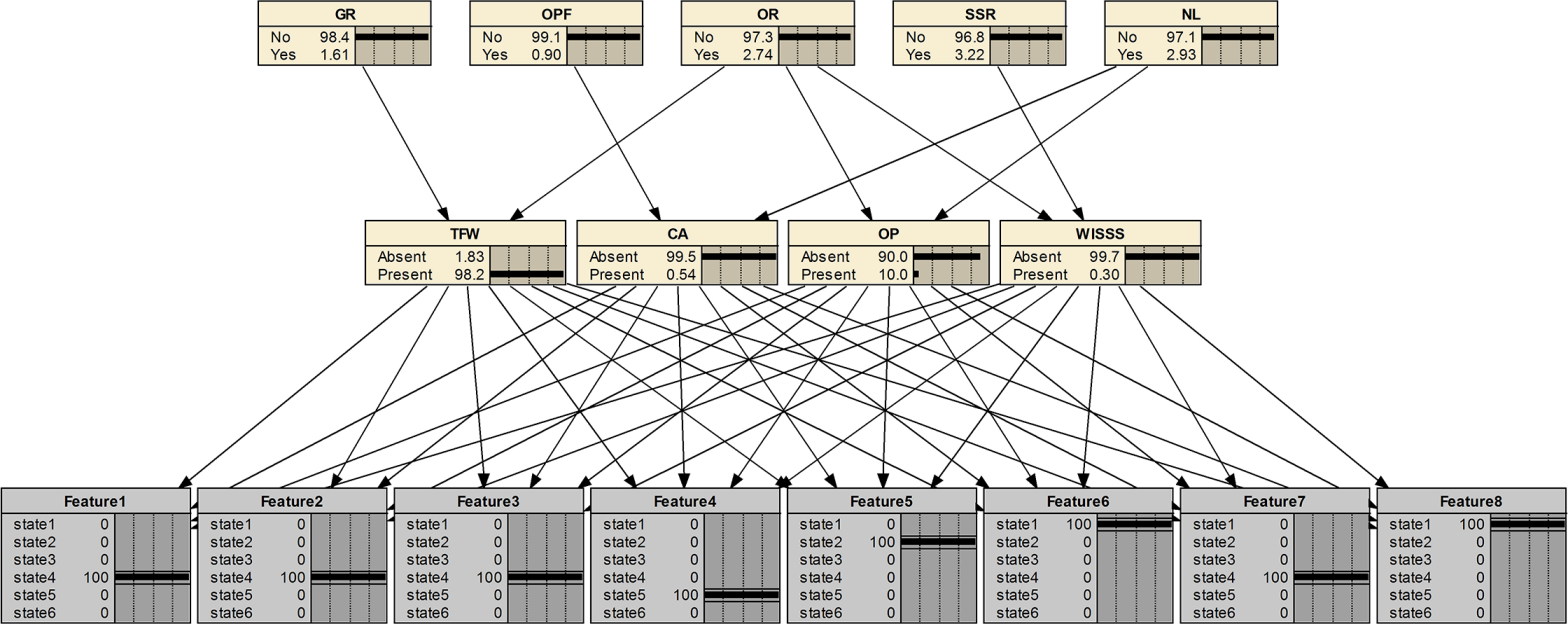
Diagnostic results of a testing sample using only fault features.

Take a feature set of TFW fault as a testing sample, T = {4, 4, 4, 5, 2, 1, 4, 1} and perform diagnosis only using these features. The diagnostic results are shown in Fig 6. It indicates that the most suspected fault is TFW (98.2%). The diagnostic result is accurate. It demonstrates that the developed Bayesian network only using the sensor data has good performance on identifying the faults.

To test the effectiveness of the model only using evidences from the fault feature layers like other researchers usually did, 200 testing instances are used. Each type of fault has 50 samples. In order to reflect the model superiority, it is necessary to build other models to compare with the proposed model. Recently, some intelligent classification algorithms, such as artificial neural network (ANN) and support vector machine (SVM) have been successfully applied to the intelligent fault diagnosis of mechanical equipment [32–34]. Features of the investigated signals are dealt with ANN or SVM to recognize the health conditions of the objects. In this paper, ANN and SVM are applied to train and test the same samples as the Bayesian network did. The test results are shown in Table 8. It demonstrates that the proposed method based on Bayesian network achieves the best diagnostic performance. Besides, the average diagnostic accuracy of ANN, SVM and Bayesian network is 94%, 95%,98.5%, respectively. The developed Bayesian network improves the average diagnosis accuracy by 4.5% and 3.5%, respectively, compared with ANN and SVM. The comparison result indicates that the proposed method outperforms the other two common methods in diagnosing different categories of gear pump faults.

**Table 8:** Testing accuracy ANN, SVM and Bayesian network.

Fault	Samples	ANN	SVM	Bayesian network
TFW	50	90%	100%	100%
CA	50	96%	100%	98%
OP	50	98%	98%	100%
WISSS	50	92%	82%	96%

### Fault diagnosis using fault features and multi-source information

**Fig 7:**
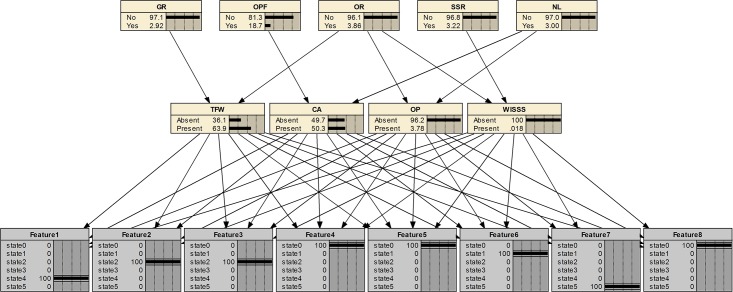
Step 1 of the fault diagnosis for the case.

**Fig 8:**
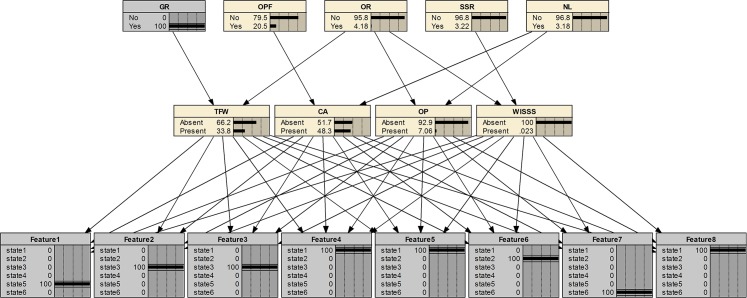
Step 2 of the fault diagnosis for the case.

**Fig 9:**
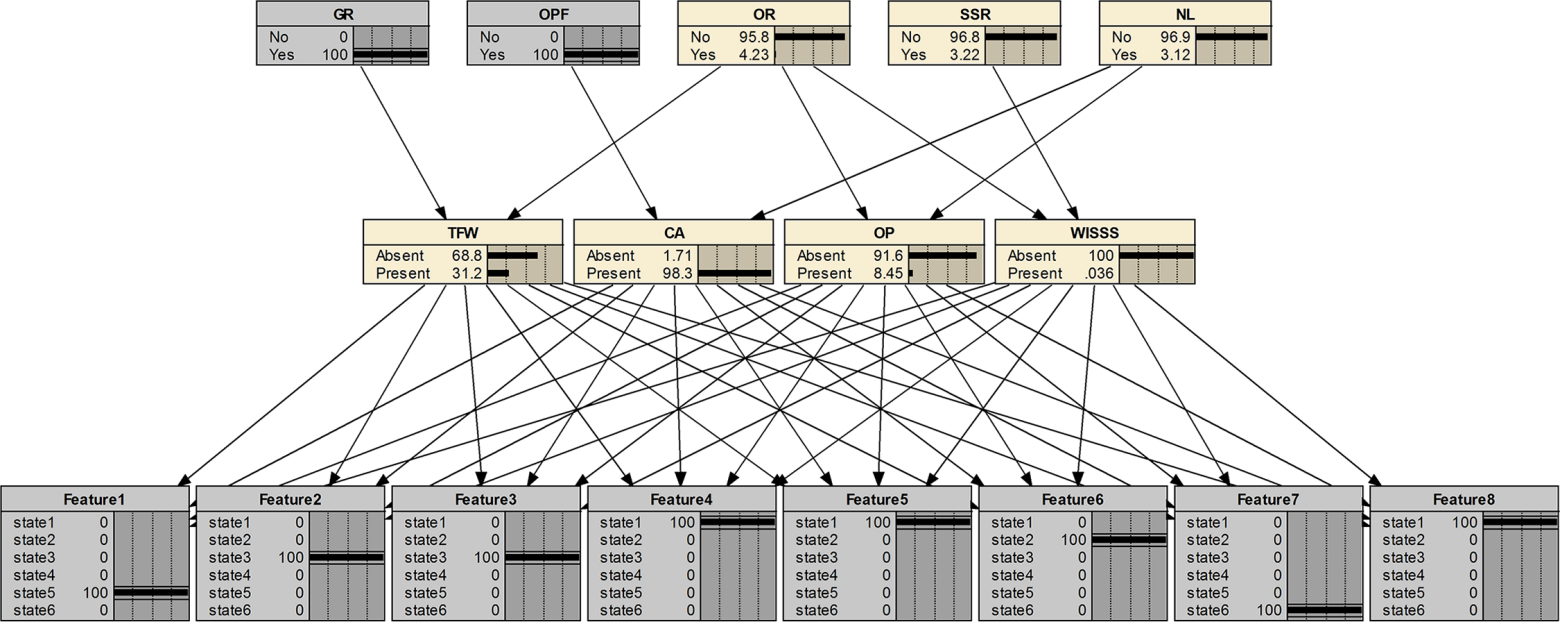
Step 3 of the fault diagnosis for the case.

Take a feature set as an example, T = {5, 3, 3, 1, 1, 2, 6, 1} and perform diagnosis using the developed Bayesian diagnostic network. The steps are shown in Figs 7–9. At step 1 in Fig 7, only fault features are applied for fault diagnosis. Probabilities of the presence of TFW, CA, OP, WISSS is 64%, 46.5%, 3.83%, 0.017%, respectively. So, it’s hard to tell whether TFW or CA occurs without other useful information. At step 2 in Fig 8, a new evidence (GR is yes) in the information layer is added. The most suspected fault is CA (48.3%). The fault probability of TFW (33.8%) is decreased under the new evidence. As shown in Fig 9, at step 3 (added evidence: OPF is yes), the fault probability of CA increases from 48.3% to 98.3%. It is because the new evidence is a unique cause for CA. It demonstrates that multi-source information is helpful to fault diagnosis.

## Conclusions

The main contribution of this paper is that a methodology based on Bayesian network and EEMD for fault diagnosis is presented. The advantages of Bayesian network and EEMD are integrated. Compared with the other conventional fault diagnosis methods, the presented methodology is able to make use of more useful information besides sensor signals. Essentially, the presented scheme based on EEMD and Bayesian network is a multi-source information fusion based methodology. With the proposed three-layered Bayesian network framework, some useful information (including naked eyes inspection, maintenance records, etc) can be helpful to identify the fault. The proposed method has been applied to the fault diagnosis of gear pump and it is effective and efficient based on vibration signals and other information.

1. The proposed methodology is applied to fault diagnosis of a gear pump. The developed diagnostic Bayesian network has three layers, namely fault feature layer, fault layer and multi-source information layer. Sensor signals and other helpful information for diagnosis could be added into the networks.
2. When fault features extracted from EEMD method are only used, the developed model has better diagnostic performance than ANN and SVM classification algorithms. It improves the average diagnosis accuracy by 4.5% and 3.5%, respectively, compared with ANN and SVM.
3. Sometimes, it may be hard to distinguish the faults only based on the sensor signals. A case study has demonstrated that some information from human observation or system maintenance records is very helpful to the fault diagnosis.
